# An Important Step

**Published:** 1882-05

**Authors:** 


### EDITORIAL.

### AN IMPORTANT STEP.

The method of preparing the dentist for the work of his profession has been, and still is, a subject of much interest, and one about which there has been, and is, considerable diversity of opinion.

In the beginning of dentistry, in this country, at least, it was regarded but little if anything more than a mere trade or handicraft. Then those wishing to enter the practice would, by hook or by crook, get a smattering of some of the processes and methods by which the ordinary operations were performed, or for a short time enter an office and gain such knowledge as the liberality (too often illiberality) of the preceptor would permit, or was able to give.

The first plan conceived of, for a liberal and systematic course of instructions for the dentist, was through the medical colleges. This was during the time from 1832 to 1838; and was first suggested and the effort made by Dr. H. H. Hayden, then of Baltimore, but it failed because the medical colleges refused to entertain any such proposition ; and so that was abandoned. But the idea of a systematic dental education was not given up, on the contrary it grew deeper and stronger as time passed on till it was wrought out in the organization and establishment of the Baltimore College of Dental Surgery, designed especially to teach dental science, art and practice.

Other similar institutions were established ere long. In the course of time the growth and outreach of the profession became such that the dental colleges, as hitherto conducted, did not meet the highest conceptions of some, at least, of the more progressive men in the profession. Notwithstanding, the dental colleges were striving to make progress, it was not such as to satisfy all; and as a result of this state of affairs, there has been, within the last ten years, six or seven dental colleges established in connection with medical colleges, under the control and management of Universities. Some advantages accrue from such a connection that cannot be realized by the dental colleges as originally organized and conducted ; and especially is this true of those institutions in which the teacher's salary is wholly independent of the fees of students. Again, schools in connection with general educational institutions can insist upon a preliminary scholarship.

A new step has been taken in Chicago on the subject of dental education. A plan has been arranged by which a full medical education, and the M. D. is to be required of each student proposing to practice dental surgery; after this is attained, or as it is attained, the student takes his special instructions from a dental didactic teacher who is a member of the medical faculty. The instruction in dental practice—clinical and prosthetic—will be given in one dental hospital which is to be thoroughly equipped and large enough to accommodate the dental students from all the medical colleges. The intention is that the dental student may enter any medical college he chooses, at least, that has made provision for him. The aim will be to give most thorough instruction in the dental infirmary and issue only a certificate of proficiency for that.

This plan is similar to that adopted in England. The profession will look with interest to this new departure.

